# Supplementary material for: Trend in Breakfast Consumption among Primary School Children in Italy
Source: Nutrients. 2023 Oct 31;15(21):4632. doi: 10.3390/nu15214632 (PMC10647676; doi:10.3390/nu15214632)
Supplement: Supplementary file 1 [file nutrients-15-04632-s001.zip › nutrients-2648497-supplementary.pdf]

Supplementary S1: Children's questionnaire- breakfast's section

Italian version and English version

1 Hai fatto colazione **questa mattina?**

☐ Sì ☐ No

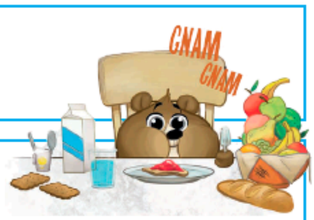

2 Se, prima di venire a scuola, hai fatto colazione, segna con una crocetta tutto quello che hai mangiato e bevuto **questa mattina:**

- ☐ latte
- ☐ latte con cacao o orzo
- ☐ tè
- ☐ succo di frutta
- ☐ spremuta
- ☐ cornetto
- ☐ brioches
- ☐ merendina
- ☐ biscotti
- ☐ panino farcito o toast
- ☐ pane e marmellata
- ☐ pane e nutella
- ☐ pizza o focaccia
- ☐ crackers o grissini
- ☐ fette biscottate
- ☐ torta o crostata
- ☐ yogurt
- ☐ cereali
- ☐ frutta
- ☐ uova

3 Se **questa mattina,** prima di venire a scuola, hai mangiato o bevuto altre cose a colazione, **oltre quelle indicate sopra,** scrivile sulla linea

____

1. Did you have breakfast this morning?

- o Yes
- o No

2. If you had breakfast before coming to school, please put a tick on everything you had this morning:

- o milk
- o biscuits
- o yoghurt
- o chocolate milk or barley
- o sandwich
- o tea
- o bread with jelly
- o a piece of fruit
- o fruit juice
- o bread with chocolate spread
- o eggs
- o fresh squeezed juice
- o pizza or focaccia
- o cereals
- o cornetto
- o crackers or bread sticks
- o brioches
- o crispy toast-like bread
- o snack
- o cake or fruit tart

3. If this morning you ate or drank something else for breakfast besides the things listed above, please make a list on the line below

____
